# Are long‐term FAD diets restricting micronutrient intake? A randomized controlled trial

**DOI:** 10.1002/fsn3.1895

**Published:** 2020-10-26

**Authors:** Neal Malik, Serena Tonstad, Michael Paalani, Hildemar Dos Santos, Wagner Luiz do Prado

**Affiliations:** ^1^ Department of Health Science and Human Ecology California State University San Bernardino CA USA; ^2^ School of Public Health Loma Linda University Loma Linda CA USA

**Keywords:** diet, high‐fiber, low carbohydrate, micronutrients, obesity

## Abstract

The micronutrient adequacy of common fad diets is rarely assessed. We compared a high‐fiber diet [HF] with a low‐carbohydrate diet [LC] to assess their effects on body weight and micronutrient adequacy. One hundred and seventy‐three adult males and females with or without diabetes with a mean body mass index of 36 kg/m^2^ were randomized to either a HF or LC diet. Differences in anthropometrics, blood lipids, glucose, blood pressure, and micronutrient consumption between groups were assessed after 52 weeks. Differences between groups a priori were assessed using independent *t* tests and chi‐squared tests. Post hoc differences in nutrient consumption between groups while controlling for gender were assessed using factorial analysis of variance. After 52 weeks, LC dieters (*n* = 24) retained weight loss better than their HF counterparts (*n* = 30) (*p* = .06). LC dieters consumed more vitamin K (mcg) [HF = 124.0 ± 15.0; LC = 220.0 ± 39.1; *p* = .025] and vitamin B12 (mcg) [HF = 3.1 ± 0.3; LC = 4.1 ± 0.4; *p* = .026]. The HF group consumed more folate (mcg) [HF = 479.9 ± 34.0); LC = 333.8 ± 22.1; *p* < .001], magnesium (mg) [HF = 353.1 ± 17.4; LC = 281.1 ± 18.0; *p* < .001], and iron (mg) [HF = 14.6 ± 0.8; LC = 10.7 ± 0.6; *p* < .001. Both groups consumed less than the respective EAR for vitamins D and E and less than the AI for potassium. While a LC diet may be more effective for long‐term weight loss, both diets were deficient in micronutrients.

## Graphical abstract

We conducted a randomized controlled trial to compare a high‐fiber diet with a low‐carbohydrate diet to assess their effects on body weight and micronutrient adequacy. Both groups consumed less than the respective RDA for vitamins D and E, magnesium, and potassium. While a LC diet may be more effective for long‐term weight loss, both diets were deficient in micronutrients.

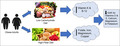

## 1 INTRODUCTION

It is estimated that 42% of the U.S. adult population are overweight or obese (Hales, Carroll, Fryar, & Ogden, 2020) and at least 33% of Americans are adhering to a restrictive diet (Brouns, 2018). Among U.S. consumers, over $60 billion are spent annually on weight loss products (Marketdata LLC, 2019). While some diets promote adherence to micronutrient recommendations, many fall short (Gardner et al., 2010).

A contributing factor to the obesity epidemic is the increased consumption of ultra‐processed foods which may contain high levels of saturated fat and added sugars, while devoid of nutrients such as protein, fiber, vitamins, and minerals (Steele, Popkin, Swinburn, Popkin, Swinburn, & Monteiro, 2017).

The pathology of obesity is multifaceted and includes behavioral, genetic, and environmental influences (Engel, Kern, Thomas Brenna, & Mitmesser, 2018). It has been reported that obesity and undernutrition are highly correlated (Weegels, 2019). Adhering to fad diets may reduce total daily calorie consumption. However, an unintended consequence may be inadequate micronutrient consumption. Micronutrient deficiencies have been found in those with obesity and may increase the risk of developing comorbidities such as diabetes, heart disease, cancer, and osteoporosis (Calton, 2010).

The popularity of low‐carbohydrate, high‐protein diets for body weight management remains high, and meta‐analyses seem to support their effectiveness for weight loss (Brehm, Seeley, Daniels, Seeley, Daniels, & D'Alessio, 2003; Hu et al., 2012; Samaha et al., 2003). This may be due to increased satiety, lipid oxidation, and energy expenditure, all of which may contribute to negative energy balance (Brehm et al., 2003). Long‐term effects of low‐glycemic, high‐fiber diets have also shown favorable effects on body weight and reduced risk of cardiovascular disease, type 2 diabetes, and colon cancer (Brouns, 2018; Reynolds et al., 2019). It has been proposed that high‐fiber diets promote weight loss through various mechanisms, including delayed gastric emptying and insulin release as well as triggering the secretion of satiety hormones such as ghrelin, glucagon‐like peptide, and polypeptide YY (Slavin, 2013). However, their respective micronutrient densities have not been adequately examined. We previously reported that a low‐carbohydrate diet was superior to a high‐fiber bean‐rich diet for body weight management over the long term, whereas those adhering to the high‐fiber bean‐rich diet experienced more favorable effects on atherogenic blood lipids (Tonstad, Malik, & Haddad, 2014). Bean consumption has been associated with a reduced risk of diabetes, heart disease, and colon cancer (Mitchell, Lawrence, Hartman, & Curran, 2009). It has been proposed that adherence to a high‐fiber plant‐based diet promotes regular consumption of antioxidants which may result in reduced systemic inflammation (Castro‐Quezada, Román‐Viñas, & Serra‐ Majem, 2014). However, analyses of the micronutrient composition of low‐carbohydrate and high‐fiber bean‐rich diets have yet to be explored. The present study served as a secondary analysis and compared a high‐fiber bean‐rich diet with a low‐carbohydrate diet with respect to micronutrient adequacy.

## 2 METHODS

### 2.1 Study design

As previously reported by Tonstad et al. (2014), a prospective, randomized controlled trial was conducted as a proof‐of‐concept study. Participants were randomly assigned to one of two diets: (a) a high‐fiber, bean‐rich diet [HF] or (b) a low‐carbohydrate diet [LC]. Women in the HF diet were encouraged to consume ≥ 40 g of fiber per day, whereas males aimed to consume ≥ 50 g of fiber per day. LC dieters were instructed to consume < 120 g of carbohydrates per day. Participants were followed for 52 weeks. Follow‐up visits were scheduled at regular intervals during the first 16 weeks and every 3 months thereafter. Participants met with a registered dietitian (RD) who conducted a 24‐hr dietary recall to monitor compliance. Three additional 24‐hr dietary recalls were conducted via phone at random between weeks 16 and 52. Changes in body weight, body mass index (BMI), waist and hip circumferences, and blood pressure were measured. Blood glucose levels, hemoglobin A_1c_ (HbA_1c_), and lipids were measured at 16 weeks and 52 weeks.

Based on Cohen's estimation of a medium effect size (*d* = 0.50), power = 80%, and *α* = .05, a total of 128 participants were needed (64 in each group) (Cohen, 1992). Accounting for a 25% dropout rate, 170 total participants were needed.

### 2.2 Participants

Individuals were recruited using flyers and informational meetings at the Diabetes Treatment Center in Loma Linda, CA. The Loma Linda University Institutional Review Board (IRB) approved the study protocol and all participants were provided written informed consent. Participants were deemed eligible if they were ≥ 18 years of age with a BMI between 30.0 and 44.0 kg/m^2^. Those with type 2 diabetes were eligible if they were clinically stable within the past 3 months. Exclusion criteria included type 1 diabetes, pregnancy, lactation, active cancer, participation in another trial, dieting within the last 3 months with weight loss exceeding 4 lbs. (1.81 kg), adherence to a vegan diet, active eating disorder, current acute or chronic infection, use of weight loss medications, or gastrointestinal problems made worse by a high‐fiber diet.

### 2.3 Procedures

**FIGURE 1:**
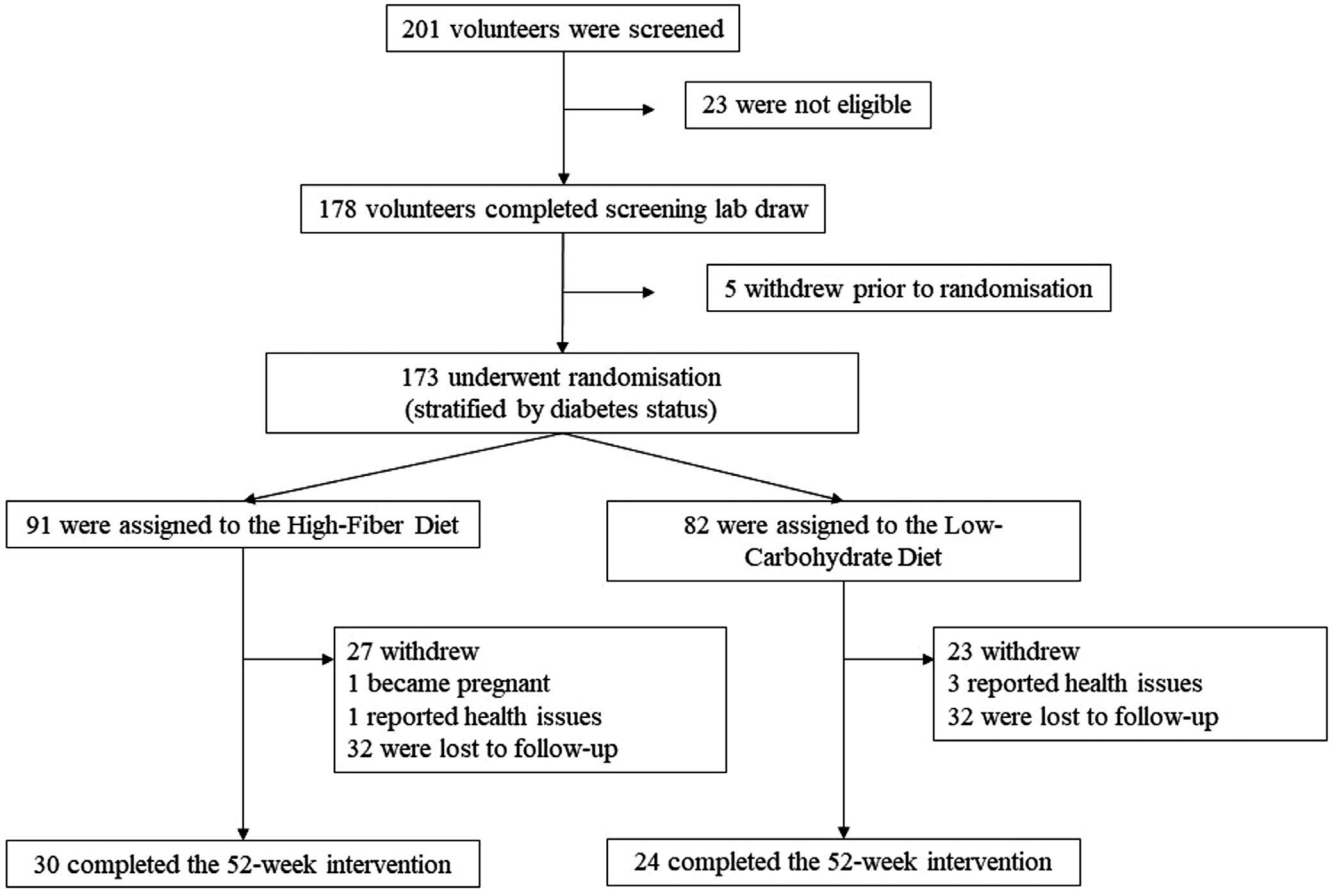
Study timeline

A total of 201 individuals were screened, in which 23 of them were ineligible. Ultimately, 173 subjects were randomized (Figure 1). A total of 91 participants were assigned to the HF group and 82 to the LC group. Married couples were randomized to the same group to improve compliance. Participants were stratified according to their diabetes status to ensure that those with diabetes were equally represented in both groups. An RD provided instructional materials (see Appendices A and B) and provided dietary education. This was followed by a 3‐week preparation phase. During this phase, those in the HF group began eating one serving (125 ml or ½ cup) of cooked beans with one meal each day. Participants were encouraged to gradually increase their dietary fiber intake by consuming one serving of cooked beans with each of three meals per day. LC dieters were encouraged to consume fewer carbohydrates while increasing their intake of protein‐rich foods such as lean meats, fish, and eggs. A 1‐week dietary induction phase followed at week 4 after baseline. Each participant was provided seven meals over the course of 3 days (two breakfasts, three lunches, and two dinners), which were distributed from the Loma Linda University Research Kitchen. Meals were consumed and were designed to educate participants with regard to portion sizes and menu options for their respective diets. Meals for the high‐fiber group provided half a cup of beans or lentils with breakfast, lunch, and dinner and emphasized whole grains, vegetables, and fruit. Meals for the low‐carbohydrate group consisted of a large meat, egg or vegetable protein portion, one or two servings of nonstarchy vegetables, and a small fruit serving.

Participants were instructed to return for maintenance visits with an RD during weeks 6, 8, 12, and 14. Additional follow‐up visits occurred at months 6, 9, and 12. During each visit, changes in body weight, BMI, waist and hip circumferences, dietary compliance, and physical activity patterns were assessed. Participants' physical activity levels were assessed using a brief questionnaire asking participants to indicate whether their physical activity levels had increased, decreased, increased vigorously, or remain unchanged since the previous visit.

### 2.4 Anthropometric measurements

Participants' body weight and height were measured without shoes. Body weight was measured using a calibrated Tanita Digital Large Capacity scale (HD‐351; Tanita Corp.), and height was assessed using a calibrated Cardinal Detecto stadiometer (Cardinal Detecto 439; Cardinal/Detecto Scale Industry Co.). Blood pressure was taken using a HoMedics Automatic Deluxe Blood Pressure Monitor (BPA‐101; HoMedics Inc.) after participants remained seated for 5 min with both feet flat on the floor. Waist and hip circumferences were determined using a Mabis DMI Healthcare Retractable Tape Measure (Mabis Healthcare, Inc.). Waist circumference was assessed at the level of the umbilicus, and hip circumference was assessed at the top of the iliac crest. Two measures were taken for each measurement and the averages were recorded.

### 2.5 Laboratory analyses

Participants were required to fast for 8 hr prior to all blood draws. Samples were analyzed at the Loma Linda University Medical Center Central Laboratory. Total cholesterol levels were assessed using the Roche Cholesterol Assay (Roche). Direct assay was used to measure LDL cholesterol, whereas HDL cholesterol used polyethylene glycol‐modified enzymes and dextran sulfate. Triglyceride levels were assessed using enzymatic calorimetric tests. The hexokinase method determined fasting blood glucose levels, and HbA_1c_ was assessed using the turbidimetric inhibition immunoassay for hemolyzed whole blood.

### 2.6 Dietary compliance

During in‐person maintenance follow‐up visits, trained research RDs conducted 24‐hr dietary recalls with each participant. Each participant was also provided with a blank food journal and encouraged to log their food and beverage intake in addition to dates, times of day, and portion sizes. These logs were also reviewed by the RDs during maintenance follow‐up visits but were not included in our data analyses. Rather, the logs were used to encourage dietary compliance. Between weeks 6–16 and months 6–12, subjects were also telephoned unannounced at random and asked to participate in a 24‐hr dietary recall to assess dietary compliance. Nutrition Data System for Research, version 2008 (2008) (Nutrition Coordinating Center, University of Minnesota, Minneapolis, MN, USA), was used to collect 24‐hr dietary recall data via telephone. Data were collected using standardized interview prompts. These data were used in our statistical analyses.

In addition to presenting data on changes in body weight, micronutrient intakes were compared between diet group and gender. Diet adequacy was determined using the established estimated average requirement (EAR) values. The following nutrients were included when determining the adequacy of the respective diets: vitamin A, thiamin, riboflavin, niacin, vitamin B6, folate, vitamin B12, vitamin C, vitamin E, magnesium, phosphorus, selenium, and zinc. It has been reported that dietary iron intake is often not normally distributed, which may lead to difficulties interpreting iron intakes (Barr, Murphy, & Poos, 2002). Therefore, iron adequacy was not assessed in our analyses. Rather, iron intakes between groups were included for comparison purposes. It should also be noted that EARs have not been established for vitamin K and pantothenic acid (IOM, 2011), and therefore, only comparisons between groups were conducted for these nutrients. EAR has not been established for potassium; however, an adequate intake (AI) cutoff has been determined (National Academies of Sciences, 2019) which was used in its place.

### 2.7 Statistical analyses

Data were entered into SPSS, version 25 (IBM, Inc.). After randomization, baseline differences between groups and changes over time were assessed using independent *t* tests and chi‐squared tests. Statistical analyses were performed regardless of whether all test assumptions are met. When test assumptions were not met, nonparametric tests were conducted and compared to the initial analyses. If *p*‐values significantly differed, the variables were transformed and re‐analyzed. Nutrient intake comparisons between groups and gender were assessed using factorial analysis of variance. Comparisons of vitamin and mineral consumption between groups controlling for gender were conducted (Table 2). Using the last observation carried forward, results for the completers are shown where *p* < .05 was considered statistically significant.

## 3 RESULTS

Of the 173 participants that were randomized, 54 completed the 52‐week intervention and data collection procedures (*n* = 24 in the LC group; *n* = 30 in the HF group). Baseline characteristics of the study sample are presented in Table 1. A higher proportion of women were enrolled in the study (74%) when compared with their male counterparts (26%). Twenty percent of those randomized were diabetic. The overall mean (±*SD*) age was 49 ± 1 years, the majority of whom identified as White (51%), followed by Hispanic/Latino (25%), Black/African American (18%), and “other” (6%). At randomization, mean (±*SD*) body weight was 99 ± 2 kg. Glucose levels differed initially between groups. This was likely due to a higher proportion of diabetics being randomly assigned to the LC group.

**TABLE 1:** Characteristics of the study population according to diet at baseline

	High fiber	Low carbohydrate	*p*‐value
	(*n* = 91)	(*n* = 82)	
	Mean (*SD*)	Mean (*SD*)	
Gender (*n* (%))			
Male	20 (22.0)	25 (30.5)	.203
Female	71 (78.0)	57 (69.5)	
Diabetics (*n* (%))	14 (15.4)	21 (25.6)	.095
Nondiabetics (*n* (%))	77 (84.6)	61 (74.4)	
Race (*n* (%))			
White	51 (56.0)	37 (45.1)	.024*
Black/African American	22 (24.2)	10 (12.2)	
Hispanic/Latino	15 (16.5)	29 (35.4)	
Other	3 (3.3)	6 (7.3)	
Age	47.7 (10.2)	49.1 (11.2)	.402
Anthropometrics			
Height (cm)	165.1 (8.9)	165.2 (8.4)	.965
Weight (kg)	100.3 (16.1)	99.2 (14.0)	.998
BMI (kg/m^2^)	36.6 (3.8)	36.3 (4.1)	.654
Systolic blood pressure (mmHg)	133 (16)	137 (22)	.153
Diastolic blood pressure (mmHg)	89 (11)	90 (14)	.739
Waist (cm)	111.6 (11.4)	112.3 (11.4)	.712
Hip (cm)	119.3 (9.3)	118.8 (9.2)	.713
Lab values			
Triglycerides (mg/dl)	140 (79)	140 (75)	.403
Cholesterol (mg/dl)	190 (34)	189 (37)	.586
HDL‐C (mg/dl)	51 (12)	50 (12)	.167
LDL‐C (mg/dl)	118 (32)	121 (33)	.416
Glucose (mg/dl)	98 (30)	106 (35)	.024*
HbA1c (%)	5.6 (0.6)	5.8 (1.0)	.147
WBC (bil/L)	6.74 (0.2)	7.37 (0.3)	.109
RBC (tril/L)	4.77 (0.4)	4.77 (0.5)	.857
Hemoglobin (g/dl)	13.6 (0.2)	13.5 (0.2)	.83
Hematocrit (%)	40.9 (0.4)	40.5 (0.4)	.731
Platelets (bil/L)	274 (8)	283 (8)	.681
Creatinine (mg/dl)	0.8 (0.02)	0.8 (0.02)	.834
TSH (uIU/ml)	2.244 (0.2)	2.535 (0.2)	.241
AST (U/L)	27 (2)	24 (1)	.425
ALT (U/L)	31 (3)	32 (2)	.927

Note

Values obtained at screening.

Abbreviations: ALT, alanine aminotransferase; AST, aspartate aminotransferase; bil/L, billions of cells per liter; BMI, body mass index; cm, centimeter^s^; g/dl, grams per deciliter; HbA1c, hemoglobin A1c; HDL, high‐density lipoprotein; kg, kilograms; LDL, low‐density lipoprotein; m, meters; mg/dl, milligrams per deciliter; mmHg, millimeters of mercury; RBC, red blood cells; tril/L, trillions of cells per liter; TSH, thyroid‐stimulating hormone; U/L, units per liter; uIU/ml, micro‐IU per milliliter; WBC, white blood cells.

* *p*‐value < .05.

As previously reported by Tonstad et al. (2014), while not statistically significant, the LC group tended to retain weight loss better than the HF group (*p* = .06), even though total energy intake and physical activity levels did not differ (data not shown).

The mean (*SD*) intakes by diet group and gender of select fat‐ and water‐soluble vitamins are presented in Table 2. Results of the statistical analyses revealed significant differences between the two groups. Expectedly, the LC group consumed higher quantities of vitamins commonly found in animal‐based products such as vitamin K and vitamin B12 (Table 2). Unsurprisingly, those assigned to the HF group consumed more folate. Mean (*SD*) differences in mineral consumption between groups and gender intakes are seen in Table 3. Somewhat expectedly, HF dieters consumed more magnesium and less calcium when compared to those in the LC group. However, quite surprisingly, these individuals consumed more iron. Of note, males and females in both groups consumed less than the EAR for vitamin D and vitamin E and less than the AI for potassium. EARs were used as the cutoff values. Recommended dietary allowances (RDAs) represent intakes that exceed the requirements of 98% of all individuals; therefore, this may overestimate the prevalence of nutrient inadequacies (Barr, Murphy, & Poos, 2002). Rather, EARs are estimates of median intakes and therefore exceed the needs of half of the sample and fall short of the needs for the other half. Because of this, EARs are considered more appropriate when assessing nutrient adequacy among groups.

**TABLE 2:** Differences in vitamin consumption according to diet and gender after 1 year (subjects with data after 1 year)

	EAR	High fiber	Low carbohydrate	*p*‐value
		(*n* = 30)	(*n* = 24)	
		Mean (*SD*)	Mean (*SD*)	
Fat‐soluble vitamins				
Vitamin A (retinol) (IU)				
Both genders		7,594.5 (1,342.2)	8,019.3 (891.6)	.808
Male	2,083	10,100.0 (19,629.6)	9,427.4 (7,906.6)	.478
Female	1,667	6,627.4 (7,094.3)	7,333.3 (6,170.9)	
Vitamin D (calciferol) (mcg)				
Both genders		2.7 (0.3)	3.1 (0.4)	.406
Male	10	3.0 (3.2)	3.9 (2.8)	.339
Female	10	2.5 (2.7)	2.7 (2.7)	
Vitamin E (alpha‐tocopherol) (mg)				
Both genders		8.1 (0.8)	7.9 (0.8)	.817
Male	12	8.8 (6.8)	10.1 (8.8)	.298
Female	12	7.9 (7.2)	6.8 (3.4)	
Vitamin K (phylloquinone) (mcg)				
Both genders		124.0 (15.0)	220.0 (39.1)	.025*
Male	–^a^	148.8 (168.5)	226.7 (287.5)	.084
Female	–^a^	114.5 (118.0)	216.8 (306.2)	
Water‐soluble vitamins				
Vitamin C (ascorbic acid) (mg)				
Both genders		93.8 (7.0)	87.6 (9.8)	.599
Male	75	82.7 (61.4)	75.8 (65,4)	.582
Female	60	98.1 (62.7)	93.4 (78.5)	
Thiamin (B1) (mg)				
Both genders		1.4 (0.1)	1.8 (0.4)	.213
Male	1.0	1.6 (0.7)	1.4 (1.1)	.380
Female	0.9	1.3 (0.6)	2.0 (3.4)	
Riboflavin (B2) (mg)				
Both genders		1.6 (0.1)	1.8 (0.1)	.153
Male	1.1	1.9 (0.8)	2.1 (0.8)	.003*
Female	0.9	1.5 (0.7)	1.6 (0.6)	
Niacin (B3) (mg)				
Both genders		19.4 (1.0)	22.2 (1.6)	.142
Male	12	22.3 (11.0)	27.3 (14.4)	.043*
Female	11	18.3 (8.3(	19.7 (10.6)	
Pantothenic acid (mg)				
Both genders		4.6 (0.2)	5.1 (0.3)	.171
Male	–^a^	5.7 (2.7)	5.8 (2.1)	.002*
Female	–^a^	4.2 (1.7)	4.7 (1.8)	
Pyridoxine (B6) (mg)				
Both genders		1.8 (0.1)	1.7 (0.1)	.529
Male	1.1	2.1 (0.9)	2.0 (0.9)	.054
Female	1.1	1.7 (0.7)	1.6 (0.7)	
Folate (mcg)				
Both genders		479.9 (34.0)	333.8 (22.1)	<.001*
Male	320	540.8 (295.7)	335.0 (137.9)	.007*
Female	320	456.4 (303.5)	333.2 (182.8)	
Cobalamin (B12) (mcg)				
Both genders		3.1 (0.3)	4.1 (0.4)	.026*
Male	2.0	3.5 (2.6)	5.0 (3.6)	.034*
Female	2.0	3.0 (2.4)	3.7 (2.4)	

Abbreviations: IU, international units; mcg, micrograms; mg, milligrams.

^a^ EARs have not been established for vitamin K or pantothenic acid.

* *p*‐value < .05.

**TABLE 3:** Differences in mineral consumption according to diet and gender after 1 year (subjects with data after 1 year)

	EAR	High fiber	Low carbohydrate	*p*‐value
		(*n* = 30)	(*n* = 24)	
		Mean (*SD*)	Mean (*SD*)	
Minerals				
Calcium (mg)				.076
Both genders		743.1 (38.3)	818.1 (51.4)	.235
Male	800	874.3 (369.9)	899.6 (450.3)	
Female	800	692.5 (317.6)	778.3 (340.6)	
Phosphorus (mg)				.966
Both genders		1,203.6 (55.5)	1,207.1 (62.7)	.002*
Male	580	1,472.7 (556.5)	1,401.6 (573.0)	
Female	580	1,099.7 (427.8)	1,112.4 (397.6)	
Magnesium (mg)				.005*
Both genders		353.1 (17.4)	281.1 (18.0)	<.001*
Male	350	431.4 (166.4)	319.3 (157.1)	
Female	265	322.8 (140.4)	262.5 (124.3)	
Iron (mg)				<.001*
Both genders		14.6 (0.8)	10.7 (0.6)	<.001*
Male	6	18.1 (7.6)	12.4 (5.7)	
Female	5	13.3 (5.9)	9.9 (4.1)	
Zinc (mg)				.403
Both genders		10.1 (0.5)	9.4 (0.6)	.001*
Male	9.4	12.5 (5.5)	11.4 (5.3)	
Female	6.8	9.1 (3.7)	8.4 (4.2)	
Copper (mg)				<.001*
Both genders		1.5 (0.1)	1.1 (0.1)	<.001*
Male	0.7	1.8 (0.8)	1.3 (0.7)	
Female	0.7	1.4 (0.7)	1.0 (0.4)	
Selenium (mcg)				.151
Both genders		90.6 (4.5)	101.7 (6.6)	.115
Male	45	105.1 (49.0)	108.4 (45.7)	
Female	45	85.0 (34.6)	98.5 (52.6)	
Sodium (mg)				.284
Both genders		2,719.8 (151.0)	2,974.5 (185.0)	.026*
Male	1,500	2,971.3 (1,052.4)	3,654.2 (1,800.7)	
Female	1,500	2,622.7 (1,435.4)	2,643.5 (1,048.3)	
Potassium (mg)				.151
Both genders		2,768.5 (138.6)	2,490.9 (121.4)	.030*
Male	3,400	3,240.4 (1,362.8)	2,734.2 (1,003.5)	
Female	2,600	2,586.4 (1,138.0)	2,372.4 (873.0)	

Note

EARs have not been established for sodium or potassium.

Abbreviations: AI, adequate intake; mcg, micrograms; mg, milligrams.

* *p*‐value < .05.

## 4 DISCUSSION

We previously reported that LC dieters had significantly higher mean intakes of total, saturated, monounsaturated, polyunsaturated, and *trans* fats and cholesterol (Tonstad et al., 2014). These analyses indicate that micronutrient deficiencies may be present in those following fad diets.

### 4.1 Vitamins

#### 4.1.1 Vitamin D

Adequate vitamin D consumption through foods may be difficult given that many common foods are poor sources (Office of Dietary Supplements, 2019b). Results revealed that both diet groups consumed less than the RDA for vitamin D. The United States Department of Agriculture (USDA, 2020) lists salmon, tuna, vitamin D‐fortified orange juice, milk fortified with vitamin D, and yogurt, as foods with high concentrations of vitamin D (defined as ≥ 20% DV). Based on the dietary recall analyses, those in both diet groups commonly consumed yogurt (all varieties) and cow's milk as their main sources of vitamin D (data not shown). While both groups did not differ in their average vitamin D consumption, all participants consumed less than the EAR (10 mcg/day) [HF = 2.7 ± 0.3 mcg/day; LC = 3.1 ± 0.4 mcg/day; *p* = .406].

Long‐term consumption of vitamin D below the EAR may increase chronic disease risk. Osteomalacia has been well established as a result of long‐term vitamin D deficiency in both children and adults (IOM, 2010a). A positive correlation between obesity and vitamin D deficiency has also been reported (Bell et al., 1985). Vitamin D is stored in body fat cells; however, it may not be bioavailable which may account for this correlation (Holick, 2004). While optimal serum concentrations of vitamin D, particularly its most active form of 25‐hydroxyvitamin D [(25(OH)D], have not been established (Cranney et al., 2007), there is speculation that adequate consumption of vitamin D may prevent type 1 and type 2 diabetes (Hyppönen, Läärä, Reunanen, Järvelin, & Virtanen, 2001; Pittas et al., 2006), hypertension (Krause, Bühring, Hopfenmüller, Bühring, Hopfenmüller, Holick, & Sharma, 1998), and cancer (Holick, 2004). Regarding cancer, it has been proposed that 25(OH)D regulates cell growth and induces cell apoptosis. However, the Agency for Healthcare Research and Quality concluded further research is needed to determine the relationship between vitamin D and health outcomes unrelated to bone health (Newberry et al., 2014). Over the long term, inadequate consumption of vitamin D‐rich foods may increase an individual's risk for developing cancer, metabolic, or skeletal diseases.

#### 4.1.2 Vitamin E

While vitamin E is found in a variety of forms, alpha‐tocopherol is considered the most significant in humans (IOM, 2000). However, the majority of dietary vitamin E is consumed as gamma‐tocopherol (Dietrich et al., 2006). Food sources with high concentrations of gamma‐tocopherol include vegetable oils, such as corn, canola oil, and soybean oils (USDA, 2020). Given its high concentration of gamma‐tocopherol, soybean oil may not be considered an adequate source of vitamin E, whereas sunflower and canola oils are considered good sources (Grilo et al., 2014). Sunflower seeds, almonds, hazelnuts, and peanut butter are also considered good sources of vitamin E (defined as ≥ 20%DV) but may not be high in alpha‐tocopherol (USDA, 2020). Commonly consumed sources of vitamin E among study participants included peanut butter (particularly among those in the LC group) and almonds in the HF group (data not shown). In contrast, NHANES 2001–2002 data estimate mean intakes between 19.3 and 24.9 mg/day among males and females 19–50 or >50 years (Gao, Wilde, Lichtenstein, Wilde, Lichtenstein, Bermudez, & Tucker, 2006).

Vitamin E is involved in immune function, cell signaling, and regulation of gene expression (Traber, 2006). It has been proposed that vitamin E protects cells against damage caused by free radicals by ceasing production of reactive oxygen species during fat metabolism. Vitamin E may also aid in the dilation of blood vessels and platelet aggregation promoting the release of prostacyclin from the endothelium (IOM, 2000). The effect of vitamin E supplementation on disease risk has been extensively studied in vitro. Based on these results, it has been proposed that vitamin E inhibits oxidation of low‐density lipoprotein (LDL) cholesterol and prevents to blood clots due to its anticoagulation effect (Glynn, Ridker, Goldhaber, Ridker, Goldhaber, Zee, & Buring, 2007). Data from NHANES 2001–2002 estimate that average vitamin E consumption from food is likely above the RDA (Gao et al., 2006). However, adherence to restrictive diets may decrease food consumption of vitamin E, particularly in the form of the alpha‐tocopherol isomer. As previously stated, our data appear to replicate these findings. Bostick et al. (1993) discovered a reduced risk of colon cancer in women with higher intakes of vitamin E from foods. Similarly, 30 IU/day (20.1 mg/day) was associated with a 20% lower risk of developing age‐related macular degeneration when compared to those with low intakes (<15 IU/day; 10.0 mg/day) (Chong, Wong, Kreis, Wong, Kreis, Simpson, & Guymer, 2007; Evans, 2007). Risk for cognitive decline, including dementia and Alzheimer's disease, has also been examined. Morris, Evand, Bienias, Evand, Bienias, Tangney, and Wilson (2002) discovered a lower risk for cognitive decline in elderly individuals that consumed higher quantities of vitamin E from foods or supplements. Adherence to a restrictive diet over the long term may lead to an increased risk for cognitive decline, macular degeneration, and certain forms of cancer.

### 4.2 Minerals

#### 4.2.1 Calcium

It has been reported that across the lifespan, females consume less dietary calcium from food (Ervin, Wang, Wright, & Kennedy‐Stephenson, 2004). The majority of study participants identified as female (74%) (Table 1). Mean intakes among females in the HF group were lower than their LC counterparts (Table 3). U.S. NHANES 2003–2006 data revealed that mean calcium intake from foods and supplements in women ranges from 918 to 1,296 mg/day (Bailey et al., 2010). However, it has been estimated that only 30% of calcium is absorbed from food (IOM, 2010b). Beginning in childhood, the absorption rate of calcium in the gut begins to decrease such that by adulthood, only 15%–20% of dietary calcium is absorbed (Office of Dietary Supplements, 2020). The absorption rate continues to decrease throughout adulthood, which partially explains the increased EAR for calcium among females older than 50 years and among males and females older than 70 years (1,000 mg/day) (Heaney, Recker, Stegman, Recker, Stegman, & Moy, 1989; NIH, 1994). However, consuming calcium in the presence of vitamin D promotes the absorption of calcium in the gut (USDA, 2020).

Good food sources of calcium (≥20% DV) include yogurt, milk, cheddar cheese, and fortified juices and beverages (such as soy milk) (USDA, 2020). Calcium deficiency may not be immediately evident given blood calcium levels are tightly regulated. However, long‐term calcium intakes below the recommended levels may result in osteoporosis (Office of Dietary Supplements, 2020) for which postmenopausal women are particularly at risk. While clinical trials do not support a correlation between calcium and loss of body weight, adequate intakes over the long term may reduce chronic disease risk, including colon and rectal cancer (Tantamango‐Bartley et al., 2017). Dairy products, which are considered good sources of calcium (≥20% DV), may also reduce blood pressure (Appel et al., 1997). However, low‐fat dairy products may be most beneficial for reducing blood pressure due to the potential atherogenic effects of full‐fat dairy.

### 4.3 Magnesium

Magnesium supports many physiological functions, including DNA synthesis, energy production, muscle contraction, and regulation of blood glucose and blood pressure (IOM, 1997; Rude, 2010, 2012). Magnesium homeostasis is closely regulated by the kidneys through modifications in urinary excretion (Office of Dietary Supplements, 2019a). Dietary magnesium can be obtained by consuming common foods, particularly those that contain dietary fiber such as spinach, nuts, seeds, and legumes (USDA, 2020). Males and females in the LC group consumed less than the respective established EAR for gender.

While excess magnesium consumption from supplements may lead to a diuretic effect, this has not been demonstrated with foods. Whole, unprocessed foods are typically higher in magnesium as the refining process may affect magnesium content (IOM, 1997). U.S. NHANES 2005–2006 data estimate that those from all age groups consumed 284 mg/day, which is less than EAR (Moshfegh, Goldman, Ahuja, Goldman, Ahuja, Rhodes, & LaComb, 2009). It has been reported that dietary magnesium intakes greater than 250 mg/day may reduce the risk for ischemic heart disease and hemorrhagic stroke (Del Gobbo et al., 2013; Larsson, Orsini, & Wolk, 2012). We found that men and women in the LC group consumed more than this cutoff on average (Table 3). Of particular interest given the study sample, meta‐analyses (Larsson et al., 2012; Schulze et al., 2007) have revealed an inverse relationship between magnesium intake and risk of type 2 diabetes, particularly among those with a BMI ≥ 25.0 kg/m^2^ (Dong, Xun, He, Xun, He, & Qin, 2011). Increased consumption of magnesium‐rich foods may be recommended for those with overweight or obesity as a potential preventive measure against metabolic disease with minimal side effects. The most commonly consumed magnesium‐containing foods among those in the HF group were almonds and legumes (beans), whereas those in the LC group likely consumed the majority of their magnesium in the form of peanut butter (data not shown). Almonds, cooked spinach, and cashews are considered good sources of magnesium (≥20%DV) (USDA, 2020). HF dieters consumed more almonds, which may partly explain the differences in consumption across groups.

### 4.4 Potassium

It has been well established that those with obesity are at an increased risk for developing hypertension (Koliaki & Katsilambros, 2013; Kotsis, Stabouli, Papakatsika, Stabouli, Papakatsika, Rizos, & Parati, 2010). The physiological mechanisms by which this occurs are complex and involve multiorgan systems. Those with obesity may experience low‐grade chronic inflammation which in turn may increase insulin resistance, endothelial resistance, and sodium retention (Kotsis et al., 2010; Ramos et al., 2003). The IOM (2005) has established an AI of 1,200–1,500 mg/day and a tolerable upper limit of 2,300 mg/day. A meta‐analysis conducted by the World Health Organization (WHO) (2012) revealed that normotensive individuals and those with hypertension that consume > 2,000 mg/day may increase their risk of developing hypertension. However, increased potassium consumption may offset this effect (Geleijnse, Kok, & Grobbee, 2003) by decreasing blood volume through vasodilation while increasing the rate of sodium excretion (IOM, 2005).

Reductions in blood pressure have been seen in those that regularly consume potassium‐rich foods such as fruits and vegetables (Champagne, 2006) which is preferred to supplementation (Appel et al., 2006). However, results from national surveys estimated that among those 20 years of age or older, mean daily potassium intakes from foods were 3,016 mg for men and 2,320 mg for women (Oria, Harrison, & Stallings, 2019), which fall below the respective AI (3,400 mg/day for males; 2,600 mg/day for females). In our study, those assigned to the HF group consumed more potassium when compared with those in the LC group. However, participants in both groups consumed less than the AI. The most commonly consumed food sources of potassium among HF dieters were beans, lentils, tomatoes, and bananas. In contrast, LC dieters regularly consumed chicken, cheese, and Greek yogurt (data not shown). Apricots are considered good sources of potassium (≥ 20%DV), whereas lentils, kidney beans, and bananas are considered moderate sources (9%–16%DV) (USDA, 2020). HF participants were encouraged to increase their intake of dietary fiber by consuming beans and lentils, which specifically may have led to the observed differences. After 1 year, participants in the HF group did not experience significant reductions in either diastolic or systolic blood pressure, despite their potassium consumption (Table 1).

### 4.5 Study strengths and limitations

The study design, including randomization of participants and its duration, were key strengths. Regular follow‐up visits with an RD and the collection of multiple 24‐hr dietary recall data clearly showed diet compliance among those in both groups. This was true despite the fact that, historically, adherence to prescribed diets among study participants is quite low (Moyad, 2005). While dietary compliance was high, attrition among the study participants was quite high (69%). A meta‐analysis revealed attrition rates for prospective weight loss studies range from 2.5% to 48.8% (Millstein, 2014). Using the last observation moved forward method allowed for adequate statistical power. A more representative sample, particularly a proportionate number of males within each diet group, may have allowed for greater generalizability. Assessments of micronutrient status via laboratory samples as well as an evaluation of nutrient deficiency symptoms should be included in future studies.

The present study served as a secondary analysis and compared a high‐fiber bean‐rich diet with a low‐carbohydrate diet with respect to micronutrient adequacy. Dieters in each group significantly differed with regard to their micronutrient intakes. Given the popularity of high‐fiber and low‐carbohydrate diets, these findings may guide health professionals and the public alike with regard to maximizing dieters' nutrient intakes during their weight loss efforts. Nutrition supplementation may be necessary. Future research should employ methods to minimize attrition and consider including more male participants in the sample. Additionally, assessments of micronutrient status via laboratory samples as well as an evaluation of nutrient deficiency symptoms should be included in future studies.

## CONFLICT OF INTEREST

The authors declare that there are no conflicts of interest.

## TRANSPARENCY DECLARATION

The lead author affirms that this manuscript is an honest, accurate, and transparent account of the study being reported. The reporting of this work is compliant with CONSORT guidelines. The lead author affirms that no important aspects of the study have been omitted and that any discrepancies from the study as planned (Loma Linda University Institutional Review Board) have been explained.

## Data Availability

Data available on request due to privacy/ethical restrictions. The data that support the findings of this study are available on request from the corresponding author. The data are not publicly available due to privacy or ethical restrictions.

## Appendix A Guide to high‐fiber diet

**Table:**

	Foods to choose	Foods not allowed
Breads, cereals, grains	Whole wheat breads (100% whole wheat or whole grain) Regular oatmeal Whole‐grain and high‐fiber ready‐to‐eat cereals (cheerios, shredded wheat, fiber 1, raisin bran, bran cereals) Brown rice Whole wheat pasta Corn tortillas Whole‐grain pancakes and waffles	White bread Refined and sugary ready‐to‐eat cereals (rice krispies, Kellogg K, trix, fruit loops) Instant oatmeal, cream of rice, cream of wheat White rice White pasta Flour tortilla Chips, crackers Pancakes and waffles made with refined flour Muffins, doughnuts, breakfast pastries, pop tarts
Vegetables	All vegetables: raw, cooked, canned, frozen Salads	Fried vegetables
Fruits	All fresh or frozen fruit Dried Fruits	Fruits canned in syrup Fruit juice
Milk and dairy foods Limit 2 servings per day	Serving size 1 cup fat‐free, 0.5%, 1% milk 1 cup soy milk 1 cup low‐fat and nonfat yogurt ½ cup cottage cheese 1 ounce low‐fat cheese	Regular cheese (Cheddar, Swiss) Pudding Ice cream
Protein foods, meats Limit Meat, chicken, or fish to 2 servings per day 1 serving = 3 oz cooked meat or the size of a deck of cards	Cooked beans (black beans, kidney beans, limas, navy beans, garbanzos) Cooked lentils and peas Tofu Soy products (meat analogs) 2 servings (3 oz per serving) a day of lean beef, chicken, turkey, or fish. Can be boiled, sautéed, or roasted. 3 eggs per week	Fried chicken Breaded or fried fish
Fats and Oils	Vegetable oil (olive, canola, soybean) Salad dressing Peanut butter Olives Avocado	Butter Margarine
Sweets	Diet drinks and soda	Cakes, cookies brownies, candy, pies

## Appendix B Guide to low‐carbohydrate diet

**Table:**

	Foods to choose	Foods not allowed
Starchy foods Limit 2 servings per day for women 3 servings per day for men	Serving size 1 slice bread 1/4th bagel ½ English muffin ½ cup cooked oatmeal ½ cup cooked rice ½ cup cooked pasta ½ cup cooked beans, lentils, or peas 1 small tortilla (4 inch) 3/4th cup ready‐to‐eat cereal	More than 2 servings per day for women or 3 servings per day for men of starchy foods
Vegetables Limit Starchy vegetables (corn, potatoes, yellow squash or carrots) to ½ cup per day	Broccoli, zucchini, green beans, mushrooms, asparagus, spinach, and other green leafy vegetables Lettuce, cabbage, and other salad greens Tomatoes Peppers, cucumbers, celery, radishes	More than ½ cup of starchy vegetables
Fruits Limit 3 servings per day	Serving size 1 medium fruit (apple, pear, banana, peach, orange) ½ cup chopped fresh or frozen fruit 1 cup strawberries or melon 1/4th cup dried fruits	Fruits canned in syrup Fruit juice
Milk and dairy foods	Fat free, 0.5%, 1% milk Soy milk, unsweetened Low‐fat and nonfat yogurt, unsweetened Cottage cheese White cheese Cheddar, Swiss, Gouda, Mozzarella cheese Eggs	Sweetened yogurt Sweetened soy drinks Ice cream
Protein foods and meats	Beef, chicken, turkey, fish Hamburger Cold meats Tofu Soy products (meat analogs) Eggs	Fried chicken Breaded or fried fish
Fats and oils	Vegetable oil (olive, canola, soybean) Salad dressing Peanut butter Olives Avocado	Butter Margarine
Sweets	Diet drinks and soda	Cakes, cookies brownies, candy, pies
